# Risk factors and epidemiologic predictors of blood stream infections with New Delhi Metallo-b-lactamase (NDM-1) producing *Enterobacteriaceae*

**DOI:** 10.1017/S0950268819000256

**Published:** 2019-03-06

**Authors:** B. M. Snyder, B. T. Montague, S. Anandan, A. G. Madabhushi, A. K. Pragasam, V. P. Verghese, V. Balaji, E. A. F. Simões

**Affiliations:** 1 University of Colorado School of Medicine, Aurora Colorado, USA; 2 Department of Clinical Microbiology, Christian Medical College Hospital, Vellore, India; 3 Department of Paediatrics, Christian Medical College Hospital, Vellore, India; 4 Department of Epidemiology, Center for Global Health, Colorado School of Public Health, Aurora Colorado, USA

**Keywords:** Bloodstream infections, carbapenem-resistant Enterobacteriaceae, Klebsiella, NDM-1, risk factors

## Abstract

Carbapenem-resistant *Enterobacteriaceae* conferred by New Delhi metallo-b-lactamase (NDM-1) resistance mechanism are endemic in India and Southeast Asia. An understanding of risk factors for NDM-1 infections is necessary to guide prevention strategies. We performed a retrospective case-control study of patients admitted at Christian Medical College Hospital, Vellore, India between May 2010 and August 2014 with *Klebsiella pneumoniae* blood stream infection (BSI). We compared patients with BSI caused by NDM-1 producing strains to two control groups: BSI with other multidrug resistant (MDR) strains and BSI with pan-susceptible strains. The study groups were assessed for risk factors for the outcomes: (1) infection with any MDR strain compared to pan-susceptible; and, (2) infection with NDM-1 strain as compared with other MDR and (3) Mortality. A total of 101 patients with BSI with NDM-1 producing *Klebsiella pneumoniae* were matched to two groups of controls: 112 with non-NDM-1 MDR strains and 101 with pan-susceptible strains. Medical (OR 10.4) and neonatal (OR 0.7) ICU admission, central venous catheter placement (CVC, OR 7.4) predicted MDR BSI. Prior carbapenem use (OR 8.4) and CVC (OR 4.8) predicted acquisition of an NDM-1 strain. Significant predictors for mortality included ICU stay (OR 3.0), mechanical ventilation (OR 3.2), female gender (OR 2.2), diabetes (OR 0.4). CVC placement, prior carbapenem use and ICU admission were significantly associated with BSI with NDM-1 producing and other MDR strains.

## Introduction

Antimicrobial resistance, particularly among *Enterobacteriaceae* is associated with worse healthcare outcomes including longer hospitalisations, greater healthcare expenditures, as well as increased morbidity and mortality [1]. Given the frequent resistance of *Enterobacteriaceae* to fluoroquinolones and extended-spectrum *β* lactamases (ESBL), carbapenems have been the treatment of choice for multi-drug resistant (MDR) gram-negative organisms. Their increasing use, however, has led to the emergence of carbapenem-resistant *Enterobacteriaceae* (CRE) strains for which available therapeutic options are limited and associated mortality is high [2, 3].

In 2008, a new mechanism of carbapenem-resistance, New Delhi metallo-b-lactamase (NDM-1) was identified in isolates from the urine of a Swedish patient admitted to a hospital in New Delhi [4]. This resistance mechanism has since been identified in a range of gram-negative organisms with evidence of both chromosomal integration and carriage in plasmids [5]. NDM-1 positive isolates have been reported worldwide, with the largest burden of cases in India and Southeast Asia [6, 7]. Based on *in vitro* testing, therapeutic options for patients with NDM producing strains are often limited to agents such as polymyxins with significant toxicity, though high dose carbapenem regimens and combination therapies have been tried [8].

Prior studies of patients with CRE have identified ICU admission, prior antibiotic use and the presence of urinary and central vascular catheters as risk factors for CRE acquisition though patients with NDM-1 comprised only a small fraction of these subjects [9–12]. A significant proportion of patients with NDM-1 producing *Enterobacteriaceae*, however, do not have identified risk factors and appear to be community-acquired [13, 14]. Potential reservoirs in the environment include public water sources, companion and food-producing animals and migratory birds [7, 15].

Given the limited therapeutic options, a better understanding is needed of the risk factors for both acquisitions of infection with NDM-1 producing strains to guide both prevention efforts and the targeting of novel therapies. In this study, we sought to investigate the characteristics, risk factors and outcomes of patients with bloodstream infections (BSI) with NDM-1 producing *Klebsiella pneumoniae.*

## Methods

### Study design and setting

This study was a retrospective case-control study of BSI with *Klebsiella pneumoniae* conducted at the Christian Medical College Hospital (CMCH), a 2858 bed tertiary care hospital serving over 2.5 million patients annually, in Vellore, South India. CMCH serves as a referral centre for patients throughout India as well as internationally. *Klebsiella pneumoniae* organisms were confirmed by routine microbiology, antimicrobial susceptibility testing and targeted sequencing. Cases were patients admitted to CMCH between May 2010 and August 2014 with a carbapenem-resistant NDM-1 producing organism, identified from a database in the Department of Clinical Microbiology. There were two contemporaneously admitted control groups. The first included randomly selected patients amongst those with pan-susceptible strains, while the second was selected from those with BSI secondary to other Non NDM-1, MDR strains including ESBL-producing bacteria and those with other resistance mechanisms.

### Bacteriology

Blood samples were drawn aseptically and inoculated into BacT/ALERT 3D bottles. On signalling positive, isolates staining gram-negative were sub-cultured on 5% sheep blood and MacConckey agars with subsequent, confirmatory biochemical identification. Antimicrobial susceptibility testing was done using standard Kirby Bauer disk diffusion methods and interpreted using the appropriate 2013 or 2014 CLSI guidelines. The antimicrobial agents tested were: cefotaxime, ceftriaxone, cefpodoxime, ceftazidime, piperacillin/tazobactam, ticarcillin/clavulanate, meropenem, imipenem, trimethoprim/sulfamethoxazole, ciprofloxacin, chloramphenicol, amikacin, gentamicin, colistimethate. Carbapenem-resistant organisms were grown on blood agar overnight and whole genomic DNA was extracted using QIAamp DNA mini kits. Multiplex PCR was used to detect the carbapenemase genes (*bla*_SPM_, *bla*_VIM_, *bla*_IMP_, *bla*_NDM_, *bla*_KPC_, *bla*_OXA−48 like_) using published primers and protocols and the amplicons were visualized in 2% agarose gel containing ethidium bromide [16–21]. Controls for genes were provided courtesy of IHMA, Inc., USA.

### Data collection

Demographic and clinical data were extracted from the electronic medical record, hospital computerized databases and clinical or microbiological charts. Data were extracted by two separate independent reviewers, cross-validated and in instances of discrepancy were reviewed with both graders to reach consensus. Abstracted data included: demographic and geographic information, clinical and laboratory findings, comorbid diagnoses (prematurity, congenital malformations, cerebral palsy, congenital heart disease, congenital neurologic disease, neuromuscular disease, chronic lung disease, hypertension, heart disease, chronic obstructive pulmonary disease, asthma, smoking, liver disease, diabetes mellitus, chronic kidney disease, HIV/AIDS, cancer, rheumatologic disease, alcoholism), microbiologic data, admitting diagnosis, admission to NICU/PICU/ICU, ward of admission, length of hospitalisation, time to first positive culture, antibiotic use within the last 180 days, hospitalisations in the preceding 2 years, surgery in 90 days prior to admission, treatments and procedures performed during hospitalisation before positive culture date including placement of continuous foreign bodies (central venous catheter (CVC), arterial catheter, urinary catheter, endotracheal tube for mechanical ventilation, nasogastric, nasojejunal or infant feeding tube), hemodialysis, type and duration of antibiotics received, clinical outcome, patient disposition (discharged alive, died, discharged against advice, transferred to another facility), readmission and readmission diagnoses.

### Categories and definitions

Ages were grouped as <1 year, 1–11 years, 12–17 years, 18–64 years and >65 years of age. Prior antibiotic use was categorised as any use, *β*-lactam use, carbapenem use and cephalosporin use. Admission and readmission diagnoses were categorised as related to infection *vs.* not. Infections were categorised as healthcare-associated if the first positive blood culture sample was collected greater than 48 h after admission. Community-acquired infections were defined as BSI with the first positive blood culture sample collected less than 48 h after admission. Admission wards were categorised as ICU, Neonatal ICU, Hematology, Pediatrics and Other for the purposes of analysis. Presence of a continuous foreign body was defined as the use of any indwelling medical device other than endotracheal tubes for mechanical ventilation that was evaluated separately. Ventilator-associated infection was defined as isolation of the same pathogen from both respiratory and blood cultures in a mechanically ventilated patient. The period of antibiotics with confirmed efficacy was defined by the earliest and latest date of administration of antibiotics to which the identified isolate was assessed to be susceptible. This duration of effective antibiotic use has been divided into 3 groups: <5 days, 5–14 days and >14 days respectively.

### Outcome measures

Two primary outcomes were assessed: (1) infection with a MDR organism (NDM-1, ESBL, or other resistance pattern) compared with infection with a pan-susceptible strain (2) infection with NDM-1 strain compared with other MDR strains. Mortality during hospitalisation and duration of admission after the time of culture were evaluated as secondary outcomes.

### Statistical methods

Data were analysed using SAS version 9.4. Categorical variables are presented using frequencies and percentages while continuous variables are presented with medians and interquartile ranges. *χ*^2^ and Fisher's Exact test were used to compare frequencies of categorical variables. Analysis of variance using SAS Proc GLM was performed for comparison of continuous variables given the unbalanced data. Multivariate logistic regression models were developed for the three outcomes. Given the skewed distribution of this data, non-parametric analysis of variance was used to evaluate differences in length of stay. Comparison of individuals infected with any MDR strains to those infected with pan-susceptible strains was made to assess for risk factors for MDR strains broadly. The comparison of individuals infected with NDM-1 positive strains to other MDR strains was made to assess for unique risk factors related to infections with NDM-1 positive organisms. Factors with a *P* ⩽ 0.2 in univariate assessments were tested in multivariate analyses. Model building was performed with the backward selection. Variables with the highest *P*-value were selected for removal. The difference in −2 log likelihood was used to test the impact of removal from the model and variables were retained if removal caused a statistically significant reduction in −2 log likelihood.

### Ethics

The Institutional Review Boards at CMCH, Vellore and the Colorado Multiple Institutional Review Board (COMIRB) approved the study.

## Results

### Patient descriptives by age group

A total of 101 patients with BSI with NDM-1 producing *Klebsiella pneumoniae* were matched to 112 controls with MDR organisms 101 patients with pan-susceptible organisms. Of the MDR organisms, 101 had an identified ESBL resistance pattern and 11 were carbapenem-resistant but were NDM-1 negative. Demographic information, prior antibiotics use and comorbidities are displayed in Table 1. Twenty-eight per cent had been previously hospitalised and 21% had received antibiotics in the prior 180 days. Greater than 50% of infections were community acquired. An admission diagnosis of infection was documented in 53%. Fifty-three per cent were admitted to an ICU. Fifteen per cent had surgery in the preceding 90 days and 42% underwent surgery during the hospitalisation. Thirty-nine per cent received mechanical ventilation. Of these, 7% experienced a ventilator-associated infection. 91% had a central vascular catheter (CVC) placed and 9% of these experienced a catheter-associated infection. Forty per cent had an indwelling urinary catheter and 53% had another continuous foreign body in place.

**Table 1.** Demographics of individuals with identified *K. pneumoniae* bloodstream infections*

	0–11 m (*n* = 45) (%)	1–11years (*n*−21) (%)	12–17 years (*n*−14) (%)	18–65 years (*n* = 207) (%)	>65 years (*n* = 27) (%)	Overall (*n* = 314) (%)
Male	29 (64)	14 (67)	10 (71)	134 (65)	20 (74)	207 (66)
**Community acquired infection**	**44 (98)**	**15 (71)**	**9 (64)**	**135 (65)**	**16 (59)**	**219 (70)**
**From Vellore**	**19 (42)**	**4 (19)**	**1 (7)**	**58 (28)**	**12 (44)**	**94 (30)**
Comorbid Diagnoses						
Prematurity	23 (50)	–	–	–	–	24 (8)
Congenital Malformations	10 (28)	1 (5)	–	–	–	11 (3)
Congenital Heart Disease	11 (24)	2 (9)	–	1 (<1)	–	14 (4)
Neuromuscular Disease	1 (2)	–	–	2 (1)	2 (7)	5 (2)
Chronic Lung Disease	–	–	–	2 (1)	7 (26)	9 (3)
Smoking	–	–	–	15 (7)	4 (15)	19 (6)
Liver Disease	–	–	–	16 (5)	1 (3)	17 (5)
Alcoholism	–	–	–	16 (8)	1 (4)	17 (5)
DM	–	–	–	46 (22)	15 (56)	61 (19)
Chronic Kidney Disease	–	–	–	26 (13)	3 (11)	29 (9)
HIV/AIDS	–	–	–	3 (1)	−	3 (1)
** Cancer**	**–**	**9 (43)**	**9 (64)**	**37 (18)**	**8 (30)**	**63 (20)**
**Prior Hospitalisation**	**4 (9)**	**8 (38)**	**5 (36)**	**61 (29)**	**11 (41)**	**89 (28)**
**Antibiotics in 180 Days Prior to Admission**	**1 (2)**	**6 (29)**	**6 (43)**	**43 (21)**	**9 (33)**	**65 (21)**
** Any** *β***-lactam**	**–**	**5 (24)**	**6 (43)**	**29 (14)**	**7 (26)**	**47 (15)**
** Cephalosporin**	**–**	**2 (10)**	**5 (36)**	**8 (4)**	**2 (7)**	**19 (6)**
Carbapenem	–	1 (5)	1 (7)	12 (6)	1 (4)	15 (5)
Colistimethate	–	–	–	6 (3)	–	6 (2)
**Admission diagnoses included infection**	**20 (44)**	**8 (38)**	**3 (7)**	**118 (50)**	**17 (59)**	**166 (53)**
**Admission to NICU, PICU, Medical ICU**	**9 (20)**	**6 (29)**	**4 (29)**	**129 (62)**	**19 (70)**	**167 (53)**
**Admission Ward**						
** Hematology**	**–**	**6 (29)**	**5 (36)**	**22 (11)**	**2 (7)**	**35 (11)**
** Medical ICU**	**–**	**–**	**2 (14)**	**9 (4)**	**2 (7)**	**13 (41)**
** Neonatal ICU**	**38 (84)**	**–**	**–**	**–**	**–**	**38 (12)**
** Pediatrics**	**6 (13)**	**10 (48)**	**5 (36)**	**–**	**–**	**21 (7)**
** Surgical**	**1 (2)**	**5 (24)**	**–**	**59 (29)**	**3 (11)**	**68 (22)**
** Other**	**1 (2)**	**–**	**2 (14)**	**126 (61)**	**20 (74)**	**139 (44)**
Surgery during prior 90 days	1 (2)	5 (24)	4 (29)	35 (17)	3 (11)	48 (15)
Surgery during hospitalisation prior to culture	16 (36)	8 (38)	5 (36)	94 (45)	9 (33)	132 (42)
Abdominal or pelvic surgery	1 (2)	3 (14)	1 (7)	13 (6)	1 (4)	19 (6)
Endotracheal Intubation/mechanical ventilation	22 (49)	5 (24)	2 (14)	80 (39)	14 (52)	123 (39)
Ventilator associated infection	–	1 (20)	–	5 (6)	2 (14)	8 (7)
Central venous catheter	43 (96)	18 (86)	10 (71)	191 (92)	24 (89)	286 (91)
Catheter culture positive	–	2 (11)	–	22 (12)	3 (13)	27 (9)
**Urinary catheter**	**3 (7)**	**4 (19)**	**1 (7)**	**103 (50)**	**16 (59)**	**127 (40)**
**Other continuous foreign body in place during hospitalisation**	**41 (91)**	**7 (33)**	**1 (7)**	**105 (51)**	**12 (44)**	**166 (53)**
Mortality	13 (29)	4 (19)	2(14)	53 (26)	9 (33)	81 (26)

*Bolded items indicate statistically significant differences between the age groups at *P* < 0.05

### Antimicrobial resistance patterns

The susceptibilities of isolates by the resistance group are presented in Table 2. In the NDM-1 group, colistimethate was assessed to be susceptible in 97% of isolates. Only 7% were susceptible to amikacin. In the other MDR group, 63% were found to be susceptible to ceftriaxone, 86% to meropenem, 89% to imipenem, 66% to amikacin and 100% to colistimethate.

**Table 2.** Frequency of antibiotic susceptibility by resistance pattern

Susceptibility	NDM (*n* = 101) (%)	Other MDR (*n* = 112) (%)	Pan sensitive (*n* = 101) (%)
Cefotaxime	–	–	100 (99)
Ceftriaxone	–	70 (63)	101 (100)
Cefpodoxime	–	1 (<1)	101 (100)
Ceftazidime	–	–	94 (93)
Piperacillin/tazobactam	1 (1)	28 (25)	101 (100)
Ticarcillin/clavulanate	–	7 (6)	93 (83)
Meropenem	2 (2)	96 (86)	101 (100)
Imipenem	2 (2)	100 (89)	101 (100)
Trimethoprim/sulfamethoxazole	1 (1)	5 (4)	67 (66)
Ciprofloxacin	1 (1)	22 (20)	97 (96)
Chloramphenicol	1 (1)	1 (<1)	92 (91)
Amikacin	7 (7)	74 (66)	101 (100)
Gentamicin	2 (2)	16 (14)	99 (98)
Colistimethate	98 (97)	112 (100)	101 (100)

### Study group characteristics

The distribution of demographic and clinical factors by resistance mechanism is presented in Table 3 with variables significant in univariate comparisons for each model (any resistance *vs.* pan-susceptible, NDM-1 *vs.* other MDR) indicated. Fifty per cent of infections with pan-susceptible strains were identified to be community-acquired as compared with 21% for those infected with NDM-1 strains and 24% for those infected with other MDR strains. Thirty-seven per cent of those with NDM-1 strains had been previously hospitalised as compared with 24% for other MDR strains and 27% for the pan-susceptible strain. Sixty-three per cent of the NDM-1 and 72% of the other MDR groups were admitted to an ICU as compared with 48% of the pan-susceptible group. Among those with an ICU stay, the median length of stay was 8 days for the NDM-1 group, 8.5 days for the other MDR group and 4 for the pan-susceptible group. Forty-seven per cent in the NDM-1 group and 45% in the other MDR group received mechanical ventilation as compared with 26% in the pan-susceptible group. Ventilator-associated infections were less common in the NDM-1 group (2%) and other MDR group (6%) as compared with the pan-susceptible group (15%). Central venous catheters had been placed in 98% of the NDM-1 group, 91% of the other MDR group and 84% of the pan-susceptible group. Among patients with a CVC, catheter cultures were more commonly positive in the NDM-1 group (10%) and other MDR group (13%) than in the pan-susceptible group (3%). Urinary catheters and other continuously present foreign bodies were more commonly present in the NDM-1 and other MDR groups compared with the pan-susceptible group.

**Table 3.** Distribution of demographic and clinical factors by pattern of identified resistance

	NDM (*n* = 101)	Other resistance (*n* = 112)	Pan-susceptible (*n* = 101)
Age			
0–11 months	17 (17%)	24 (21%)	5 (5%)
1–11 years	9 (9%)	8 (7%)	6 (6%)
12–17 years	3 (3%)	9 (8%)	6 (33%)
18–65 years	63 (62%)	99 (86%)	72 (71%)
>65 years	9 (9%)	9 (8%)	12 (12%)
Male (*n* = 226)	64 (63%)	77 (69%)	66 (65%)
Community acquired infection* **	21 (21%)	34 (30%)	50 (50%)
From Vellore*	25 (25%)	32 (29%)	37 (37%)
Comorbid diagnoses			
Prematurity	5 (5%)	15 (13%)	4 (4%)
Congenital malformations	5 (5%)	6 (5%)	–
Congenital heart disease	7 (7%)	5 (4%)	1 (1%)
Neuromuscular Disease	2 (2%)	2 (2%)	1 (1%)
Chronic lung disease***	3 (3%)	2 (2%)	4 (4%)
Smoking*	8 (8%)	4 (4%)	7 (7%)
Liver Disease*	3 (3%)	6 (5%)	7 (7%)
Alcoholism***	4 (4%)	7 (6%)	6 (6%)
Diabetes mellitus*,**	12 (12%)	23 (21%)	26 (26%)
Chronic kidney disease	9 (9%)	7 (6%)	13 (13%)
HIV/AIDS	2 (2%)	–	1 (1%)
Cancer	22 (22%)	19 (17%)	22 (22%)
Prior hospitalisation	37 (37%)	27 (24%)	27 (27%)
Antibiotics in 180 days prior to admission*	26 (26%)	22 (20%)	17 (17%)
Any *β*-lactam	7 (7%)	7 (6%)	7 (7%)
Cephalosporin	4 (4%)	9 (8%)	4 (4%)
Carbapenem	8 (8%)	1 (<1%)	6 (6%)
Colistimethate	6 (6%)	1 (<1%)	–
Surgery during prior 90 days*	19 (19%)	18 (16%)	11 (11%)
Surgery during hospitalisation prior to culture	45 (45%)	50 (45%)	37 (37%)
Abdominal or pelvic surgery*	12 (12%)	4 (4%)	3 (3%)
Admission ward*	–	–	–
ICU	7 (7%)	5 (4%)	1 (1%)
Neonatal	12 (12%)	21 (19%)	5 (5%)
Surgical	27 (27%)	24 (21%)	17 (17%)
Pediatrics	8 (8%)	9 (8%)	4 (4%)
Other	47 (46%)	53 (47%)	74 (73%)
Admitted ICU during hospitalisation*	64 (63%)	81 (72%)	48 (48%)
Median days (IQR)	8 (4, 20)	8.5 (3.5, 14)	4 (2, 11)
Endotracheal intubation/mechanical ventilation*	47 (47%)	50 (45%)	26 (26%)
Ventilator associated infection	1 (2%)	3 (6%)	4 (15%)
Central venous catheter*,**	99 (98%)	102 (91%)	85 (84%)
Catheter culture positive	10 (10%)	14 (13%)	3 (3%)
Urinary catheter*,**	50 (50%)	45 (40%)	32 (32%)
Other continuous foreign body in place during hospitalisation**	55 (54%)	42 (38%)	28 (28%)
Antibiotic started at time of culture			
Colistimethate	11 (11%)	5 (4%)	4 (4%)
Tigecycline	4 (4%)	–	–
Cefoperazone-sulbactam	2 (2%)	9 (8%)	4 (4%)
Amikacin	5 (5%)	6 (5%)	3 (3%)
Other Aminoglycoside	2 (2%)	2 (2%)	1 (1%)
Meropenem	11 (11%)	32 (29%)	17 (17%)
Pipercillin-tazobactam	5 (5%)	19 (17%)	16 (16%)
Ciprofloxacin	2 (2%)	2 (2%)	7 (7%)
Antibiotic used with confirmed susceptibility of isolate	85 (84%)	2 (2%)	46 (46%)
Days antibiotics with confirmed efficacy			
Less than 5	85 (84%)	62 (55%)	48 (48%)
Died	32 (38%)	15 (29%)	11 (23%)
Median hospital length of stay (IQR)	20 (9, 41)	12 (4, 29)	11 (4, 20.5)
Median length of stay after culture (IQR)	9 (3, 20)	6(3, 14)	4.5 (1, 12.5)
5–14	9 (9%)	36 (32%)	45 (45%)
Died	1 (10%)	6 (17%)	3 (7%)
Median hospital length of stay (IQR)	18.5 (14, 24)	19 (14.5, 30.5)	15 (11, 19)
Median length of stay after culture (IQR)	13 (6, 24)	14 (10, 19)	11 (8, 16)
Greater than 14 days	7 (7%)	14 (13%)	8 (7%)
Died	1 (17%)	4 (31%)	1 (11%)
Median hospital length of stay (IQR)	42 (33, 52)	34 (26, 43)	36 (30, 47)
Median length of stay after culture (IQR)	38 (20, 41)	28 (20, 34)	32 (23, 37)
Overall mortality ***	34 (33%)	32 (29%)	15 (15%)
Median hospital length of stay (IQR) ***	21 (10, 41)	19 (11, 32)	14 (6, 25)
Median length of stay after culture (IQR)	10 (4, 20)	12 (4.5, 20)	10 (3, 16)
Discharged Alive	58 (57%)	73 (65%)	78 (77%)
Readmitted <30 days fever or sepsis	10 (18%)	3 (4%)	2 (3%)
Discharged against medical advice (*n* = 21)	7 (7%)	4 (4%)	8 (8%)
Transferred to another hospital (*n* = 6)	2 (2%)	3 (3%)	–

*Evaluated in model selection for multivariate model for any resistance *vs*. pan-susceptible based on *P* < 0.2.

**Evaluated in model selection for multivariate model for NDM-1 *vs*. other MDRO based on *P* < 0.2.

***Significant with *P* < 0.05.

### Antibiotic usage by study group

The initial antibiotic prescribed at the time of the culture varied, with colistimethate and meropenem being most common in the NDM-1 group (11% for each); meropenem (29%) and piperacillin-tazobactam (27%) in the other MDRO group; and meropenem (17%) and piperacillin-tazobactam (16%) in the pan-susceptible group. Eighty-four per cent of the NDM-1 group, 55% of the other MDRO group and 48% of the pan-susceptible group received less than 5 days of antibiotics with confirmed susceptibility. Nine per cent in the NDM-1 group, 32% in the other MDR group and 45% in the pan-susceptible group received 5–14 days of antibiotics.

### Length of stay and readmission

The median length of stay was statistically significantly longer (*P* < 0.01) in the NDM-1 group (21 days) and other MDR group (19 days) as compared to the pan-susceptible group (14 days). Length of stay after the positive culture was not significantly different between the resistance groups. Of those discharged alive, 18% in the NDM-1 group, 4% in the other MDR group and 3% in the pan-susceptible group were readmitted with a diagnosis of fever or sepsis.

### Mortality

Mortality percentages by demographic and clinical factors are summarised in Table 4. Among those who died, 42% were infected with a NDM-1 strain, 39% another MDRO organism and 19% a pan-susceptible strain. Fifty-five per cent were men and 23% had a community-acquired infection, 80% were admitted to an ICU, 68% had received mechanical ventilation and 90% had a central venous catheter placed.

**Table 4.** Distribution of demographic and clinical factors by mortality status*

	Discharged alive (*n* = 237) (%)	Died (*n* = 77) (%)	*P*-value
Age			0.55
0–11 months	33 (14)	13 (17)	
1–11 years	18 (8)	4 (5)	
12–17 years	13 (5)	2 (3)	
18–65 years	155 (65)	49 (64)	
>65 years	18 (8)	9 (12)	
**Male**	**164 (69)**	**41 (53)**	**0.01**
**Female**	**73 (31)**	**36 (47)**	
**Resistance pattern**			**<0.01**
** NDM-1+**	**67 (28)**	**34 (44)**	
** Other multidrug resistant organism**	**84 (35)**	**28 (36)**	
** Pan-susceptible**	**86 (36)**	**15 (19)**	
**Community acquired infection**	**78 (33)**	**19 (25)**	**0.17**
Comorbid diagnoses			
** Diabetes mellitus**	**52 (22)**	**8 (10)**	0.04
** HIV/AIDS**	**0 (0)**	**3 (4)**	**0.01**
Alcoholism	15 (6)	2 (3)	0.26
Chronic lung disease	5 (2)	4 (5)	0.23
Chronic kidney disease	24 (10)	5 (6)	0.28
Prior hospitalisation	65 (28)	24 (30)	0.53
Antibiotics in 180 days prior to admission	49 (21)	16 (20)	0.71
**Surgery during prior 90 days**	**41 (17)**	**8 (10)**	**0.12**
Surgery during hospitalisation prior to culture	95 (40)	35 (45)	0.37
**Admission ward**			**0.19**
** Hematology**	**31 (13)**	**6 (8)**	
** ICU**	**5 (2)**	**6 (8)**	
** Neonatal**	**28 (12)**	**10 (13)**	
** Surgical**	**49 (21)**	**17 (22)**	
** Pediatrics**	**19 (8)**	**4 (5)**	
** Other**	**105 (44)**	**34 (44)**	
**Admitted ICU during hospitalisation**	**103 (43)**	**61 (79)**	**<0.01**
**Endotracheal intubation/mechanical ventilation**	**69 (29)**	**51 (66)**	**<0.01**
**Central venous catheter**	**210 (89)**	**75 (97)**	**0.02**
**Urinary catheter**	**85 (36)**	**39 (51)**	**0.02**
**Other continuous foreign body in place during hospitalisation**	**110 (46)**	**54 (70)**	**<0.01**
Days of antibiotics with confirmed susceptibility of isolate			n/a
Less than 5 days duration	133 (56)	61 (79)	
5–14 days	80 (34)	10 (13)	
Greater than 14 days	24 (10)	6 (8)	

*Bolded elements were evaluated in model selection for multivariate model for mortality based on *P* < 0.2 in univariate assessment

### Independent predictors of outcomes

The results of the multivariate models are presented in Table 5. In the multivariate model of any resistance *vs.* pan-susceptible, the strongest predictors of infection with a resistant strain were: placement of a CVC (OR 7.4), ICU admission (OR 10.4), NICU admission (OR 7.7) and placement of a urinary catheter (OR 2.4). Though individually not significant predictors, the variables abdominal or pelvic surgery and receipt of antibiotics in the last 180 days were retained in the model due to the improved performance of the model with their retention based on the log-likelihood score. In the multivariate model of NDM-1 *vs.* other MDR, the strongest predictors were: prior carbapenem use (OR 8.4) and placement of a CVC (OR 4.8).

**Table 5.** Multivariate analysis of predictors of multidrug resistance, NDM-1 positivity and mortality*

Outcome	Reference	Predictor	Estimate
Any multidrug resistance	Pan sensitive	**Central vascular catheter**	**7.4 (1.3–39.6)**
		**Urinary catheter**	**2.4 (1.2–4.8)**
		Hematology admit	2.2 (0.8–6.4)
		**ICU admit**	**10.4 (1.2–92.0)**
		**NICU admit**	**7.7 (2.3–26.4)**
		Pediatric admit	3.7 (0.9–14.1)
		Surgical admit	2.0 (0.9–4.5)
		Abdominal or pelvic surgery	0.3 (0.1–1)
		Antibiotics in the past 180 days	2.4 (1.0–5.4)
NDM-1 positive	Other resistance	**Prior carbapenem use**	**8.4 (1.0–68.1)**
		**Central vascular catheter**	**4.8 (1.0–22.5)**
Death	Survival	**ICU stay during hospitalisation**	**3.0 (1.5–5.9)**
		**Mechanical ventilation**	**3.2 (1.7–6.0)**
		**Female**	**2.2 (1.2–3.8)**
		**Diabetes**	**0.4 (0.2–0.9)**

*Bolded parameters were significant with *p* < 0.05.

Significant predictors for mortality included ICU stay (OR 3.0), mechanical ventilation (OR 3.2), female gender (OR 2.1). Diabetics had lower odds of mortality (OR 0.4).

## Discussion

This study provides an evaluation of risk factors for the development of NDM-1 positive and MDR *K. pneumoniae* bloodstream infections in a highly endemic setting in Vellore, India. Though infections with MDR organisms are typically associated with hospital-acquired infections, it is notable that greater than 21% of NDM-1 and 24% of other MDR infections were found to be community-acquired.

The high proportion of cases with catheter-associated infections in this population is notable and this offers a potential target for reducing the risk of bloodstream infections. The risk of BSI associated with CVC catheters has been well documented and evidence-based prevention strategies exist. Though gram-positive bacteria are more commonly associated with the central line-associated BSI (CLABSI), in a prior series from India gram-negative organisms were present in as much as 36% of cases and *Klebsiella* sp were specifically identified in 8% [22]. In the same series, gram-negative organisms were more frequent in cases associated with local infection, as was placement at a femoral site. In a smaller case-control study in South Africa, central vascular catheter placement was a significant risk factor for health care-associated infections with NDM-1 positive organisms [12]. Further studies in this context examining the extent to which local contamination of the line either by site or as part of line care contributes to the rate of CLABSI with MDR strains and with NDM-1 producing organisms specifically may be useful [23].

Admission to a medical or neonatal ICU was an important predictor of infection with resistant strains as was the admission ward overall with a trend towards higher risk of resistant strains also seen in admissions to hematology, surgery and pediatrics. The identification of high-risk units is important to guide potential interventions to prevent infection. In a fecal surveillance study of ICU patients in New Delhi, carriage of carbapenem-resistant *Enterobacteriaceae* was found in 18.1% with 9.4% of them carrying NDM-1 positive strains. In studies of ICU patients in India and Tunisia, acquisition of antibiotic-resistant strains was demonstrated during admission with duration of admission and antibiotic use being predictors [24, 25]. The significant proportion of community-acquired infections may suggest that fecal carriage of MDR strains may be present prior to admission.

The mortality we observed was lower than in both a prior series of patients with CRE BSI and a smaller case-control study of healthcare-associated infections with NDM-1 positive organisms [10, 12]. Mortality was similar between patients with NDM-1 positive infections and patients with infections with other MDRO. It is possible that this reflects the underlying illness associated with the acquisition of either category of infection. It is notable, that a significant number of individuals with BSI with NDM-1 positive and other MDRO survived despite receiving less than 5 days of confirmed effective antibiotics. In some of these cases patients were treated with antibiotics such as cefoperazone for which susceptibility testing was not available. Because this was a retrospective review, it was not possible to determine the rationale for not extending therapy beyond 5 days of antibiotics with confirmed efficacy in cases where shorter durations were noted. In cases with good clinical outcomes, it may be that the decision was made based on the patient's clinical response to treatment.

Discordance between *in vitro* susceptibilities and clinical outcomes have been previously described for patients infected with NDM-1 positive organisms [26]. Animal model data have suggested that the efficiency of carbapenem hydrolysis by NDM-1 may not be sufficient to completely overcome the inhibitory effect of carbapenems and continuous-infusion and high dose regimens may retain activity [27]. *In vivo* resistance may additionally reflect the combination of both the presence of carbapenemases and other resistance mutations leading to the variability in clinical outcomes seen across studies. Mortality, however, remains significant among patients admitted with infections with MDR strains and strategies to prevent these infections are needed.

## Limitations

Given that this was a retrospective, case-control, study, it is not possible to determine the absolute risk associated with the identified risk factors. Data available were limited to that recorded in the medical record and associated databases. This may be particularly relevant with regard to the assessment of the risk associated with care received prior to hospitalisation, documentation of which may have been incomplete.

## Conclusions

This study highlights that infection with NDM-1 and other MDR strains is associated with significantly increased mortality. We identified important risk groups for infection with NDM and other MDR *K. pneumoniae* bloodstream infections that can be used to guide future research on strategies for reducing infection risk.
